# Survey of *Legionella* Species Found in Thai Soil

**DOI:** 10.1155/2012/218791

**Published:** 2012-01-12

**Authors:** Tatiana C. Travis, Ellen W. Brown, Leonard F. Peruski, Duangkamon Siludjai, Possawat Jorakate, Prasert Salika, Genyan Yang, Natalia A. Kozak, Maja Kodani, Agnes K. Warner, Claressa E. Lucas, Kathleen A. Thurman, Jonas M. Winchell, Somsak Thamthitiwat, Barry S. Fields

**Affiliations:** ^1^Respiratory Diseases Branch, Division of Bacterial Diseases, National Center for Immunization and Respiratory Diseases, Centers for Disease Control and Prevention, 1600 Clifton Road, Mailstop G03, Atlanta, GA 30333, USA; ^2^International Emerging Infections Program, Thailand Ministry of Public Health-U.S., Centers for Disease Control and Prevention Collaboration, Department of Disease Control, Ministry of Public Health, Tivanond Road, Nonthaburi 11000, Thailand

## Abstract

Members of the Gram-negative genus *Legionella* are typically found in freshwater environments, with the exception of *L. longbeachae*, which is present in composts and potting mixes. When contaminated aerosols are inhaled, legionellosis may result, typically as either the more serious pneumonia Legionnaires' disease or the less severe flu-like illness Pontiac fever. It is presumed that all species of the genus *Legionella* are capable of causing disease in humans. As a followup to a prior clinical study of legionellosis in rural Thailand, indigenous soil samples were collected proximal to cases' homes and workplaces and tested for the presence of legionellae by culture. We obtained 115 isolates from 22/39 soil samples and used sequence-based methods to identify 12 known species of *Legionella* represented by 87 isolates.

## 1. Introduction

Legionellosis is most often attributed to inhalation of contaminated aerosols from manmade water systems or, in the case of *Legionella longbeachae*, to inhalation of contaminated potting mixes or composts [1–3]. From 2003 to 2004, active population-based surveillance for atypical bacterial respiratory pathogens was performed in the province of Sa Kaeo, Thailand, to establish incidence [4]. Immunologic testing performed on sera of suspect legionellosis cases found 20/397 (5%) adult cases exhibited a fourfold rise in titer to *L. longbeachae* by the indirect immunofluorescence assay. *L. longbeachae* infection followed the typical legionellosis demographic, with incidences highest among adults over 34 years of age, increasing steadily with age, and infection peaking through September and October. As rice farming is quite prevalent in this area of Thailand, the extensive exposure of farm workers to moist soil would be a plausible means for transmitting *L. longbeachae* [5]. These findings, coupled with limited access to composted or processed soil, led us to investigate the presence of legionellae, specifically *L. longbeachae*, in indigenous soils and exposure due to agricultural practices in Sa Kaeo.

## 2. Materials and Methods

In 2009, thirty-nine wet soil samples were collected from eight rural sites within Sa Kaeo province where prior laboratory-confirmed cases of legionellosis were identified and were shipped to the Centers for Disease Control and Prevention in Atlanta for culture [4]. Cultures were performed using a modification of the procedure used to culture legionellae from water [6]. Five grams of soil were weighed, and mixed with 50 mL sterile dH_2_O. The filtrate was then strained through sterile gauze into a 50 mL conical tube. A 1 : 5 dilution was made of the filtrate, and 500 *μ*L of the diluted filtrate was acid treated for fifteen minutes with an equal part of KCl/HCl acid (pH 2.3). After acid treatment, 50 *μ*L was cultured on one plate of buffered charcoal yeast extract (BCYE) supplemented with 160 mg/L cycloheximide, two plates additionally supplemented with 100,000 U/L polymyxin B and 5 mg/L vancomycin (PCV), and two plates further supplemented with 2 g/L glycine (GPCV). Plates were examined at four, seven, and fourteen days to check for the presence of *Legionella*. Any samples overgrown with non-*Legionella* organisms were retreated with acid in fifteen-minute increments and recultured, until nonrelevant organisms were reduced enough to allow for identification of legionellae. Colonies displaying *Legionella* morphology were checked for cysteine auxotrophy on BCYE biplates with and without L-cysteine. Colonies requiring L-cysteine for growth were considered presumptive *Legionella* species.

Monoclonal antibody (MAb) testing was used to identify *L. pneumophila* serogroup 1 (Lp1) as previously described [7, 8]. Other *L. pneumophila *serogroups were determined using direct fluorescent antibody staining performed on dried, formalin-fixed suspensions using *L. pneumophila *serogroup-specific fluorescein isothiocyanate labeled antibody [9]. Select non-*L. pneumophila* isolates were tested by slide agglutination as described [10].

All non-Lp1 isolates were identified by sequencing the macrophage infectivity potentiator (*mip*) gene [11]. The resulting sequence was then queried against the National Center for Biotechnology Information (NCBI) GenBank nucleotide database using NCBI's alignment tool Basic Local Alignment Search Tool (BLAST) [12, 13]. Sequences with a minimum of 95% identity to a known *Legionella* species were assigned. Those sequences with less than 95% identity to sequences of submitted *Legionella* species were considered a potential novel *Legionella *organism. All *L. pneumophila* isolates were genotyped using the sequence-based typing (SBT) epidemiological typing scheme established by the European Working Group for *Legionella* Infections (EWGLI) as previously described [14, 15]. eBURST analysis was performed to observe the relatedness of the soil isolates to isolates from other countries previously submitted to the EWGLI SBT database (http://eburst.mlst.net/).

## 3. Results and Discussion

Twenty-two (56%) of the 39 soil samples received were positive for *Legionella*. In total, we obtained 115 isolates, 87 of which were known species, 25 potential novel species, and 3 isolates that were not typable by *mip* sequencing (Table 1). Nine of the species identified have been reported in association with human disease and represented 70% (80/115) of isolates obtained [16]. Isolates of *L. birminghamensis*, *L. lansingensis*, *L. pneumophila*, *L. rubrilucens*, and *L. sainthelensi* were found exclusively in soil samples from the personal residence of suspect cases. Isolates of *L. bozemanae*, *L. erythra*, *L. gormanii*, *L. quateirensis*, *L. quinlivanii*, and the potentially novel species identified as *Legionella* sp. ST24644 (NCBI accession number GU083740; isolated from a cooling tower in Thailand) were found only in soil samples from the workplace of suspect cases. No isolates of *L. longbeachae *were identified. The highest diversity of species (*n* = 7) was found in soils taken from the outdoor area for washing at the personal residence and within rice fields. *L. pneumophila *serogroup 1 was identified in the environment; however, none were positive for monoclonal antibody MAb2, the phenotypic subtype responsible for 65–100% of Lp1-caused legionellosis [17]. 

Five of 112 isolates tested reacted strongly with *L. longbeachae* serogroup 1 antisera by direct fluorescent antibody testing. Sequence analysis, however, indicated these isolates were *L. bozemanae *(*n* = 2), *Legionella *sp. ST24644 (*n* = 2), and a novel nonfluorescent *Legionella* species (*n* = 1). Although the species of the three *mip* untypable isolates remain unknown, they were identified as *Legionella* spp. using a pan-*Legionella *real-time PCR assay [18]. Slide agglutination testing found these isolates were not *L. geestiana*, a species in which the *mip *gene is known to not amplify with the primers used [11].

Eight allelic profiles were identified by SBT analysis, seven of which were novel profiles (as of November 2, 2010). Two of the seven novel sequence types identified were found to be related to isolates from community-acquired and nosocomial cases through eBURST analysis (as of December 14, 2010; data not shown). The eighth allelic profile matched the existing ST260 which has been associated with community-acquired cases.

Although a primary goal in this study was to identify the environmental source(s) of the suspect causative agent *L. longbeachae *in the 2003/4 suspect pneumonia cases, we were unable to recover this species from these 39 soil samples. Interestingly, the water-saturated soils collected did support the growth of many other *Legionella *species not previously associated with this indigenous soil type.

These findings suggest that the *L. longbeachae *seropositive-pneumonia cases documented in 2003/4 were either (i) due to *L. longbeachae* that were no longer or never present in these environments or were not found in our limited number of samples as mentioned previously; (ii) due to a serologically cross-reactive strain of legionellae; (iii) serological cross-reaction with another pathogen; or (iv) false-positives. Cross-reactivity is an inherent problem in the use of serology for identification, and the findings of this study further highlight the need for a molecular-based method for identification [19–22]. When possible, a clinical isolate is preferred for diagnosis of suspect non-*L. pneumophila* cases because of insufficient specificity and sensitivity of non-*pneumophila *legionellae antisera [23].

For future studies, we wish to conduct prospective surveillance in Sa Kaeo and obtain legionellae isolates from patient samples. A clinical isolate would allow for sequence identification of the etiologic agent and detection of any novel species responsible for respiratory disease in tropical countries such as Thailand. Concurrent environmental sampling would allow identification of settings capable of transmitting these pathogens to susceptible hosts. Although *L. longbeachae* is widely accepted as the predominant pathogenic, soil-dwelling species of *Legionella, *the presence of legionellae in soils has been limited to composts and manufactured potting mixes. The findings in this study indicate native soils are a likely reservoir of multiple *Legionella* species in regions with a geography and climate similar to Sa Kaeo and may play a role in human disease. 

## Figures and Tables

**Table 1:** Isolates obtained and their geographic distribution.

	Personal residence											Workplace						
Area proximal to soil sample	Temple artesian well	Temple outdoor showers	Pump water	Vegetable garden	Tank water	Outdoor area for dish washing	Pool	Front garden	Outdoor shower	Area behind house	Artesian well	Rice field	Sugarcane plantation	Lotus pond	Cattle pen	Cattle pasture	Orchard	Total

*L*. *anisa* ^a^				1		9					1	6			4			21
*L*. *birminghamen*sis^a^					1	2												3
*L*. *bozemanae* ^a^															6			6
*L*. *erythra* ^a^															1			1
*L*. feeleii^a^				1		1					16	1			4			23
*L*. *gormanii* ^a^												3						3
*L*. *lansingensis* ^a^											5							5
*L*. *pneumophila* SG1 ^a^						2												2
*L*. *pneumophila* non-SG1^a, b^		1								3	11							15
*L. quateirensis*												4						4
*L. quinlivanii*												1						1
*L. rubrilucens*						1					1							2
*L*. *sainthelensi* ^a^						1												1
*Le* *gi* *on* *el* *la* sp. ST24644^c^															4			4
Novel NF *Legionella* spp^c^				2	1	3			3		3	2			2			16
Novel BW *Legionella* spp^c^					1	1	1					2						5
*mip* untypable								1				1			1			3

Total	0	1	0	4	3	20	1	1	3	3	37	20	0	0	22	0	0	**115**

^a^Species/serogroups previously associated with human disease.

^b^ *L*.  *pneumophila*  serogroups 3, 5, and 6 present among the seven isolates tested by direct fluorescent antibody testing.

^c^ Potential novel species queried against NCBI BLAST on June 29, 2011. NCBI accession numbers JN383394 to JN383418.

Serogroup (SG), nonfluorescent species (NF), and blue-white autofluorescent species (BW).
